# Food and Its Influence on Health and Disease

**Published:** 1843-04-01

**Authors:** 


### Food and its Influence on Health and Disease. By *Matthew Truman,* M.D. 8vo. Lond. Murray, 1842. pp. 231.

This book, as is stated in the Preface, is intended more for the general reader than the medical practitioner; and it is accordingly no more than a cursory view of the extensive subject of which it treats, written in a popular style. The author, after a short introduction defining the nature of food generally and enumerating its proximate principles, proceeds to describe succinctly each of the different articles of diet belonging to the animal and vegetable kingdoms. This part of the work displays some research; as not only all the ordinary (and extraordinary) materials of nourishment made use of in our own part of the world are mentioned, but also those adopted by the inhabitants of most other regions of the globe; though it cannot be said to add much to our previous stock of knowledge on the subject. In the progress of it, however, many judicious remarks are made. For instance, in treating of milk, Dr. Truman says,—

"The care that Nature has taken to provide for the young of animals this delightful aliment, which possesses such valuable properties, that alone it is capable of affording them sufficient nourishment, during the period of their existence in which they are most helpless, ought to act upon us as a powerful incentive to ensure that the young of our own species should never be deprived of this, to them, invaluable treasure; and further, that they should always derive it, if possible, from the source which Nature intended them to procure it from. The physiologist cannot own without regret, how very commonly ladies in affluent circumstances neglect that most important of their maternal duties—the nursing of their own children. Weakness of constitution is generally pleaded as the reason for this dereliction; but the real cause is to be found in the disinclination of the gay mother to renounce the frivolities of fashionable life. Females, in general, are not aware how likely they are to injure their own health, as well as that of their infants, by refusing to subject themselves to the trouble of nursing. Hundreds might have escaped the agonies of cancer, and of many other equally serious constitutional disorders, if they had only practised the self-denial necessary for suckling their own children. * * * * An eloquent modern writer, in speaking on this subject, observes, that if he possessed a city, he would have a statue of a mother suckling her infant placed in the centre of it, as the emblem of domestic happiness!" 31-37.

But besides what ought to be had for food, Dr. Truman often gives us a dissertation on what ought not; and the worthy author would seem to gloat over the fact almost with the relish of a connoisseur when, at p. 43, he tells us—

"Human flesh is said to have the flavour of pork, and to be as tender as veal."

Nay,—(for it must be a bad cause indeed, in defence of which nothing can be urged) even if we were disposed to indulge in such tit-bits, we could, according to our author's own shewing, excuse ourselves as only following in the wake of some of our revered ancestors and treading in their steps,—

"St. Jerome says that some of the inhabitants of Britain were cannibals; and in the reign of Henry I., the Scots are stated to have killed and eaten some English, at Galloway." 44.

But let those whose "bowels yearn over us" not promise themselves too much. Whatever changes in diet we may inculcate we shall be particularly anxious not to encourage a public leaning *too strongly* towards *ourselves*, for fear of sharing the fate of our medical brethren in Egypt in the thirteenth century. At that period, we are told,—

"All sorts of stratagems were had recourse to for the purpose of entrapping people, *to which physicians were particularly exposed;* for persons pretending to be sick, sent for them under pretence of asking their advice, but really with the object of killing and eating them."

This is "cupboard-love" with a vengeance!

We hardly expect, however, that the public taste, fickle and capricious as it notoriously is, will ever return to these lengths, nor even to the diet of "the golden days of good Queen Bess"—

When ladies fair with ruffs tied round about their necks fast,
Would gobble up a pound of beefsteaks for their breaks-fast.

But we have, at the same time, no desire to verge so far from this last solid satisfying and *ration*-al kind of food as to practise living on the bark-bread of the Norwegian and Lapland peasants,—the ligneous fibres of the pine, beech, birch, elm, lime and poplar, which when ground, dried, and sifted so as to form an impalpable powder, have been "found to afford much nourishment;"—or to be obliged to avail ourselves of,

"the experiments of Autenrieth of Tubingen, and others, for making bread of sawdust mixed with a small portion of flour, which, as Herschel has remarked, deserve a higher degree of celebrity than they have obtained, because they prove that absolute famine may be rendered next to impossible." 48.

Fancy has, no doubt, a powerful influence on the human mind. But Swift has shown that my Lord Peter was unable to make his brothers, Martin and John, fancy their dry crust part of a leg of mutton, notwithstanding that the *Pope's-eye* was staring them in the face. We have an impression that we should be equally difficult of persuasion as to the identity of clay with butter.

"The German workmen at the mountain of Kiffhôusen are stated to spread clay instead of butter on their bread: they call it stein-butter, and find it satisfies their hunger like other food, and is very easy of digestion. * * * * The inhabitants of Capua are related to have formerly paid a tribute to the Neapolitans for a kind of earth called leucogæum, which they made use of in the preparation of a dish named alica. The banks of the Mackenzie river, a few miles above the Bear Lake, contain layers of a kind of unctuous mud, which the Indians in that neighbourhood eat occasionally during seasons of scarcity, and also take it even at other times for an amusement."

De gustibus non est disputandum.

Dr. Truman does not always confine himself to the relation of facts, but occasionally goes on to give their rationale. This he does in the instance last quoted. He satisfactorily shows that, under certain circumstances, clay may contain much available nutriment.

"All edible earths most likely contain portions of organic matter, which is the reason of their being taken as food. Such, at all events, has been found by Professor Retzius to be the case with the bergmehl, or flour of the mountain, at Degerfors, on the frontiers of Lapland. * * * According to Retzius, it contains the remains of nineteen different forms of infusoria with siliceous carapaxes, several of which are similar to those belonging to some of the animalculæ met with in a living state near Berlin. The earth-worm swallows large quantities of moist earth, which always has minute particles of animal and vegetable substances mixed with it, and this small quantity of nutriment is sufficient for the subsistence of the creature. Many marine animals, echinodermata, fish, &c., seem to feed almost exclusively on sand, but then that sand abounds in fragments of shells, which have been reduced to powder by the rolling of the waves on the shore. All these facts show how parsimonious Nature appears to be of organic matter, since such great care is taken that none of it shall be wasted." 67.

After sections on spices and bitters, the author treats of salt. Here he says,

"Particular care should be taken to prevent children eating their food without salt. * * * Almost all persons who abstain from it are troubled with worms." 75-6.

An adequate space is devoted to water and fermented liquors and their effect on the economy. Here occasionally some new facts or well-expressed ideas are elicited. Dr. Truman says,—

"The inhabitants of hot climates are generally supposed, though erroneously, to drink a greater quantity of water than those of cold ones. People living in the temperate parts of the earth certainly drink less than the residents of the tropics; but the inhabitants of the hyperborean regions consume a larger quantity of water than those of the hottest countries. Captain Lyon mentions that the Esquimaux drink water in enormous quantities; by gallons at a time, and two quarts at a draught." 84.

A knowledge of cookery is justly said to be "a kind of information which has more influence on the well-being of the human race than is generally imagined. The most important changes produced in the food by cookery are, the destruction of its vitality, an indispensable condition for its digestion; the coagulation of the albumen, and the liquefaction of the gelatine, osmazome, and adipose matter. These alterations, which render it more tender, sapid, and juicy, are mostly produced by exposure to a high temperature; and therefore, the observation made by Evenus, that fire is the best sauce in the world, is perfectly correct."

In speaking of cookery, Dr. Truman gives us many glimpses of the past, and recals to us that how much soever the moderns may boast of their Ude or their Jarrin, the fame of these heroes must grow pale before that of the *cuisiniers* of the Ancient Romans.

"Their cooks were so dexterous, they could serve up a pig or boar, broiled on one side, and roasted on the other. Another favourite way of dressing a boar was that of stuffing it with thrushes, beccaficoes, &c. the whole being steeped in the choicest wines and the richest gravies." 102.

Some of their dishes, indeed, would hardly suit Guildhall on the 9th of November, or the Clarendon Hotel. For instance,—

"Sausages made of sow's liver, chopped up with fat and various herbs, accompanied with a sauce of milk, or dormice smeared with honey, and sprinkled with poppy juice, would probably not be much relished at our dinners. The *altilia sumena,* considered a great delicacy, was composed of the gravid uterus of swine." 122.

Gustus elementa per omnia querunt, says Juvenal of the Romans, and assuredly

"The tocsin of the soul, the dinner bell,"

has by no means lost any of its magic power in the lapse of 1,800 years. The head cook at one of the West-end club-houses enjoyed, as we are told, at one time a salary of £1,500. a-year—a larger stipend than that of any professor in our Universities! Cardinal de Retz wisely enough warned politicians to risk no motion in a popular assembly immediately before dinner—a text, on which the walls of St. Stephen's, if gifted with speech, might utter many a commentary.

The relative influence of animal and of vegetable diet on the human frame is ably exposed—still in a strictly popular manner—by our author; who afterwards shows, by a comparison of the inhabitants of Europe with the natives of many other regions of the globe, aided by an appeal to the anatomical character of the digestive organs in man—that a mixed diet is in all respects the best suited for the full development of the body. On this head he states:—

"Many persons consider that the human race has degenerated since the variety of our food has been so much increased......... One trivial circumstance may be mentioned to prove the incorrectness of such an opinion. At the time of the tournament lately given by the Earl of Eglintoun at his seat in Scotland, when the old armour of so many departed Templars was brought out for the use of the knights who were to figure at that entertainment, many periodical publications teemed with paragraphs asserting that a great deal of padding and filling-up was necessary, to enable our young but degenerate aristocracy, (as they were called,) to keep on the corselets, arm-pieces, and sheaves, on account of the gigantic stature of the persons for whom they had originally been made. Mr. Pratt of Bond-street, who provided a large portion of this armour, states the very reverse to have been the case; that the reason, in almost every instance, the old armour had to be altered was, that it was too small instead of being too large. Most of the cuirasses and the coverings for the limbs were found to be so tight across the chest and round the arms and legs, that they could not be worn; and scarcely any of the helmets could be got on before they were enlarged: most satisfactorily proving that the higher orders of young men, in this country, possess a more perfect corporeal development than those of a similar class had six or seven hundred years ago." 140.

In the course of the work many other vulgar errors are combated. Thus, at page 63, "Sugar is supposed by many persons to be injurious to the teeth; but this is not the case, except when eaten in such excess as to cause indigestion. It is antiseptic, and preserves vegetable and animal substances from putrefaction."

At page 107, "Many are of opinion that animal food is not rendered more digestible by being cooked. Cookery, however, *does* render food easier of digestion,"—by the necessary destruction of its vitality.

A liberal allowance of nutriment is recommended in most cases by Dr. Truman, who cannot be called a "starving doctor." At page 162 he says,

"Dieticians often exclaim against the practice of giving children pies and puddings, which they consider are invariably unwholesome. This is a mistake: if a child is in a very robust state of health and can easily digest moderate quantities of those articles of diet, they are very proper for him, because they prevent his eating too much meat; and it is clear that, whenever a very large quantity of food is taken, Nature intended that it should not be of too concentrated a description."

We do not agree in every notion with our author. Thus, at page 50, he says "Wheat (*triticum*) appears to have been first found in the valley of the Jordan, on the Mountains of Lebanon, and in the parts of Palestine and Syria near Arabia." If by this it be intended to imply that Judea is *exclusively* the native land of this member of the cerealia; we must protest that such an idea is purely hypothetical. The staff of life is doubtless first alluded to in Phœnicia, it being the country in which letters originated. But if we also may indulge in an hypothesis, it is that wheat (as well as the vine, see Genesis ix, 20) is a native of the country immediately South of the Caucasus, the focus of the great Caucasian race of mankind; and that it was brought into Palestine and Egypt with the southward march of population after the Deluge. And Dr. Truman himself tacitly admits this hypothesis, in saying, that the part of the world from which the Caucasian race has sprung, "is exactly that in which all the kinds of vegetable and animal food are indigenous which seem best fitted for effecting the development of the body...... Allowing them (the Caucasians) to possess a superior natural organization, the probability must still be admitted, that they are also in a great measure indebted for their advancement before their fellow-men to their having had their origin in the region of the earth which naturally produces the most excellent kinds of nutritious matter."

A few useful rules for diet and the prevention of disease are laid down; and the terminating section is devoted to a popular view of the function of nutrition. On the whole, we have read the work with satisfaction. It is, as it professes to be, a book addressed to the public generally; and with laudable taste it is accordingly not overloaded with professional disquisitions; technical terms are also carefully eschewed throughout. No arbitrary dogmas find a place in its pages; and it has the great merit, (which many popular medical treatises cannot claim,) of being wholly free from the empiricism of recommending itself as an infallible guide in all cases—not only those in which disease prevails to a comparatively trifling extent, but those also which demand the vigilant attention of the physician.

